# Na^+^[Me_3_NB_12_Cl_11_]^−^·SO_2_: a rare example of a sodium–SO_2_ complex

**DOI:** 10.1107/S2056989019004663

**Published:** 2019-04-09

**Authors:** Carsten Jenne, Valentin van Lessen

**Affiliations:** a Anorganische Chemie, Fakultät für Mathematik und Naturwissenschaften, Bergische Universität Wuppertal, Gaussstr. 20, 42119 Wuppertal, Germany

**Keywords:** boron cluster, crystal structure, sodium, sulfur dioxide, weakly coordinating anion

## Abstract

In the title compound, Na^+^[Me_3_NB_12_Cl_11_]^−^·SO_2_ [systematic name: sodium 1-(trimethylammonio)­undeca­chloro-*closo*-dodeca­borate sulfur dioxide], the SO_2_ mol­ecule is *η* ^1^-*O*-coordinated to the Na^+^ cation. Surprisingly, the SO_2_ mol­ecule is more weakly bound to sodium than is found in other sodium–SO_2_ complexes and the SO_2_ mol­ecule is essentially undistorted compared to the structure of free SO_2_. The Na^+^ cation has a coordination number of eight in a distorted twofold-capped trigonal prism and makes contacts to three individual boron cluster anions, resulting in an overall three-dimensional network. Although the number of known *η* ^1^-*O*-coordinated SO_2_ complexes is growing, sodium-SO_2_ complexes are still rare.

## Abstract 2

In the title compound, the SO_2_ mol­ecule is *η* ^1^-*O*-coordinated to the Na^+^ cation. The Na^+^ cation has a coordination number of eight in a distorted twofold capped trigonal prism and makes contacts to three individual boron cluster anions resulting in an overall three-dimensional network.

## Chemical context   

Liquid sulfur dioxide is a polar but only very weakly coordinating solvent (Waddington, 1965 ▸), which is frequently used in organic and inorganic synthesis. The coordination chemistry in and of sulfur dioxide has been the topic of various reviews (Mingos, 1978 ▸; Ryan *et al.*, 1981 ▸; Mews *et al.*, 2000 ▸). Initially, only *S*- and *η* ^2^-*S*,*O*-coordination of SO_2_ with soft transition-metal centers were investigated, but it was subsequently shown that *η* ^1^-*O*-coordination of SO_2_ is preferred with hard main-group and transition-metal cations. Theoretical studies established that the oxygen–metal cation bonds are purely ionic (Decken *et al.*, 2009 ▸; Derendorf *et al.*, 2010 ▸). Mews and co-workers crystallized metal hexa­fluoro arsenates *M*[AsF_6_] (*M* = alkaline-earth and transition-metal cations) from liquid sulfur dioxide to obtain their SO_2_ complexes (Mews *et al.*, 2000 ▸). Unfortunately, the alkali-metal hexa­fluoro arsenates, *M*[AsF_6_] (*M* = Li, Na, K), are almost insoluble in liquid sulfur dioxide and the corresponding SO_2_ complexes remained elusive. Until recently, only two examples of alkali-metal–SO_2_ complexes were known; namely, the *η* ^2^-*O*,*O* bridged coordination complexes, [Li(OSO)_6/2_][AlCl_4_] (Simon *et al.*, 1980 ▸) and [Na(OSO)_1.5_][AlCl_4_] (Peters *et al.*, 1982 ▸), crystallized in the presence of the [AlCl_4_]^−^ anion. Only after the introduction of modern weakly coordinating anions into sulfur dioxide coordination chemistry could alkali-metal sulfur dioxide complexes be studied intentionally. By using a large fluorinated aluminate anion, the crystal structure of [(OSO)_2_Li{AlF(Al(O*R*)_3_}Li{Al(O*R*)_4_}] [*R* = C(CF_3_)_3_] (Cameron *et al.*, 2010 ▸) was determined. In addition, use of halogenated *closo*-dodeca­borates [B_12_ *X*_12_]^2−^ (*X* = F–I) led to a systematic study of alkali-metal sulfur dioxide complexes (Derendorf *et al.*, 2010 ▸). Halogenated *closo*-dodeca­borates belong to the growing class of modern weakly coordinating anions (Knapp, 2013 ▸). The [Me_3_NB_12_Cl_11_]^−^ anion represents a recent modification of the halogenated *closo*-dodeca­borates and possesses a reduced charge of −1 (Bolli *et al.*, 2014 ▸). This anion has been utilized very recently to stabilize a variety of reactive cations in the solid state (*e.g*. Bertocco *et al.*, 2016 ▸) and has been applied in silver-free gold catalysis (Wegener *et al.*, 2015 ▸). From a failed attempt to prepare [Et_3_SiOS(H)OSiEt_3_][Me_3_NB_12_Cl_11_]^−^, we obtained single crystals of the title compound as a by-product. Na^+^[Me_3_NB_12_Cl_11_]^−^·SO_2_ is a rare example of a sodium–SO_2_ complex, and its crystal structure is discussed herein.
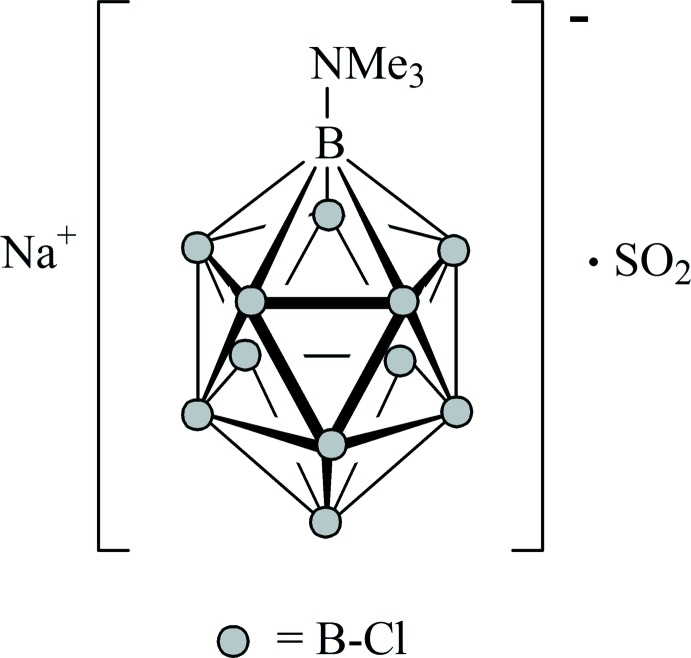


## Structural commentary   

**Figure 1:**
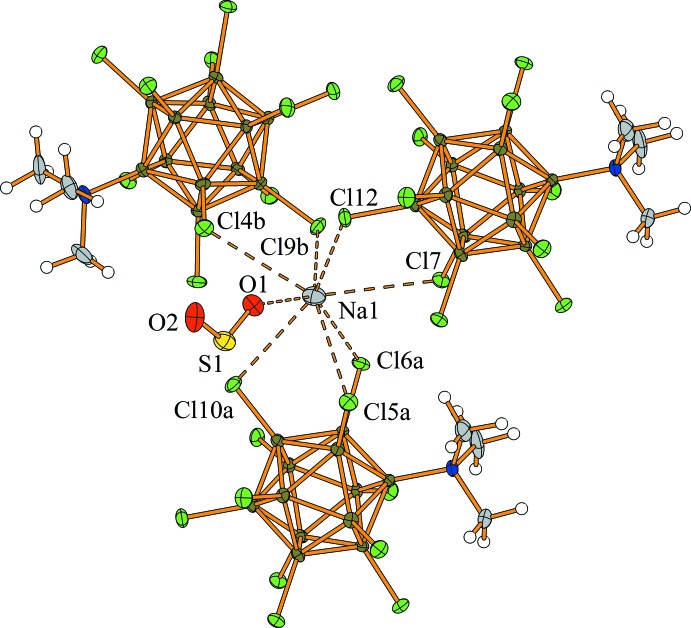
Coordination sphere around the Na^+^ cation. The Na^+^ cation makes a total of eight contacts (dashed lines) to three individual boron cluster anions and to one sulfur dioxide mol­ecule. Displacement ellipsoids are drawn at the 50% probability level and hydrogen atoms are shown with arbitrary radii. Symmetry codes: (*a*) 1 + *x*, *y*, *z*; (*b*) −1 + *x*, −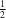 + *y*, 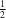 − *z*.

The title salt crystallizes with one SO_2_ mol­ecule per formula unit (Fig. 1 ▸). The SO_2_ mol­ecule is *η* ^1^-O-bonded to the Na^+^ cation, as is expected for SO_2_ coordination to hard-metal centers. The Na^+^—O distance of 2.428 (4) Å is about 0.1 Å longer than the average Na^+^⋯O distance (of 2.34 Å) found in the Na_2_[B_12_ *X*_12_]·*n*SO_2_ (*X* = H, Cl–I) complexes, which indicates weaker coordination. The S—O bonds are essentially of equal length [1.428 (3) and 1.412 (4) Å; Table 1 ▸] and very close to the values found in a free SO_2_ mol­ecule in either the solid state [1.4299 (3) Å; Grabowsky *et al.*, 2012 ▸] or in the gas phase [1.4343 (3) Å; Holder & Fink, 1981 ▸]. This behaviour is in contrast to that observed in other coordination compounds with *η* ^1^-*O*-coordinated terminal SO_2_ ligands (*e.g*. Mews *et al.*, 2000 ▸) where lengthening of the S—O_*c*_ bond and shortening of the S—O_*t*_ bond occurs, as predicted by theoretical concepts (Decken *et al.*, 2009 ▸; Derendorf *et al.*, 2010 ▸). Thus, the current finding is in accord with weaker coordination of the SO_2_ mol­ecule to Na^+^ than is found in other SO_2_ complexes of hard metal cations. In addition to coordinating to the SO_2_ mol­ecule, each sodium cation coordinates to seven of the eleven chlorine atoms of the boron cluster (Fig. 1 ▸).

**Figure 2:**
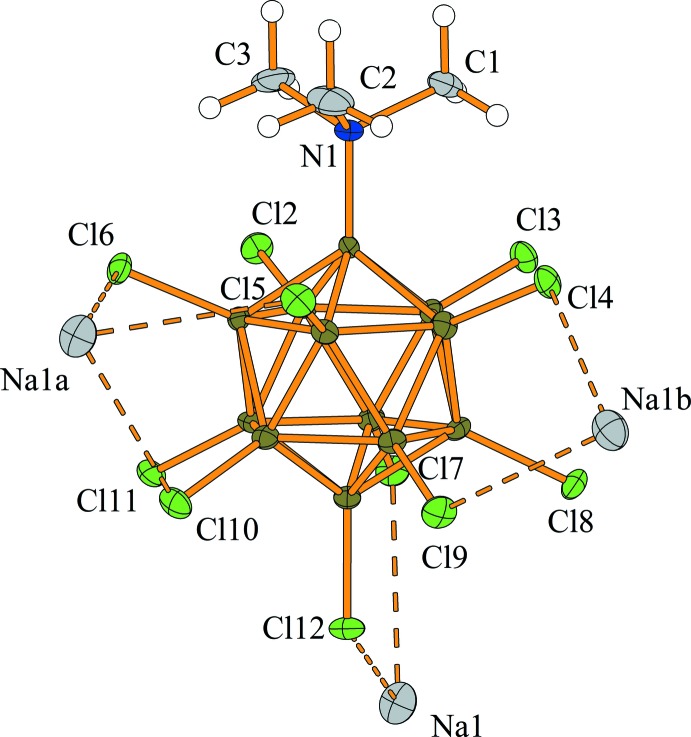
Coordination sphere around one [Me_3_NB_12_Cl_11_]^−^ anion. The boron cluster anions form a total of seven contacts (dashed lines) to three individual Na^+^ cations. Displacement ellipsoids are drawn at the 50% probability level and hydrogen atoms are shown with arbitrary radii. Symmetry codes: (*a*) 1 + *x*, *y*, *z*; (*b*) 1 − *x*, 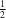 + *y*, 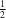 − *z*.

In the packed structure, two sodium cations are coordinated in an *η* ^2^-fashion to two chlorine atoms, while a third sodium cation is coordinated in an *η* ^3^-manner to three chlorine atoms (Fig. 2 ▸). The Cl⋯Na^+^ distances range from 2.870 (2) to 3.209 (2) Å, and are, on average, longer than those in Na_2_[B_12_Cl_12_]·4SO_2_ (*i.e.* 3.052 *vs* 2.929 Å) (Derendorf *et al.*, 2010 ▸), and are in accord with the sum of the van der Waals radius of chlorine (1.75 Å; Mantina *et al.*, 2009 ▸) and the ionic radius of sodium (1.18 Å; Shannon, 1976 ▸) of 2.93 Å. However, when anisotropy of the van der Waals radius (Batsanov, 2001 ▸) is taken into account, the inter­molecular distances are still in the expected range. The B—Cl bond lengths of the chlorine atoms coordinating to Na^+^ lie in the range 1.796 (5) to 1.803 (4) Å (av. 1.799 Å) and are only slightly longer than those of the non-coordinating chlorine atoms [1.779 (5) to 1.797 (4) Å, av. 1.786 Å]. It has previously been noted that the presence of strong Lewis acids, such as Me^+^ or *R*_3_Si^+^, leads to a significant elongation of the B—Cl bonds by up to 0.1 Å (Bolli *et al.*, 2010 ▸, 2014 ▸; Kessler *et al.*, 2010 ▸). Therefore, in the title compound, the Cl⋯Na^+^ inter­action can be classified as weak and the singly charged [Me_3_NB_12_Cl_11_]^−^ anion is more weakly coordinating towards Na^+^ than the doubly charged [B_12_Cl_12_]^2−^ anion.

## Supra­molecular features   

**Figure 3:**
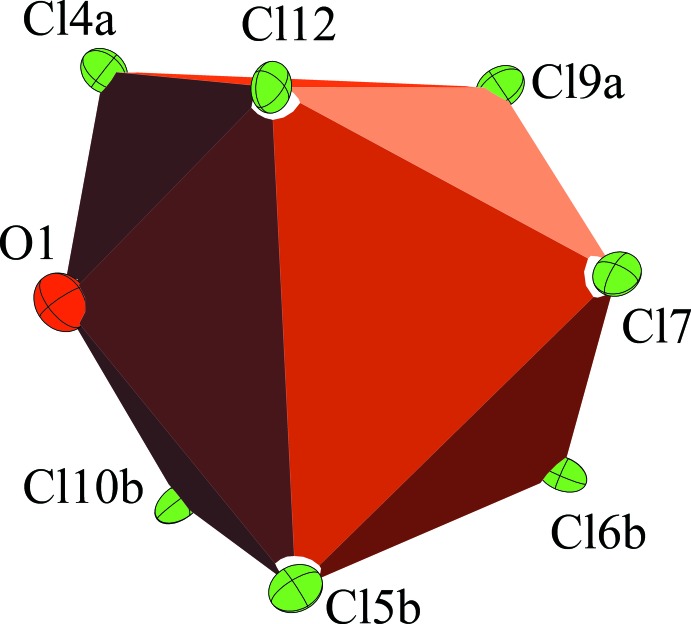
Distorted twofold-capped trigonal prism around the Na^+^ cation. Displacement ellipsoids are drawn at the 50% probability level.

**Figure 4:**
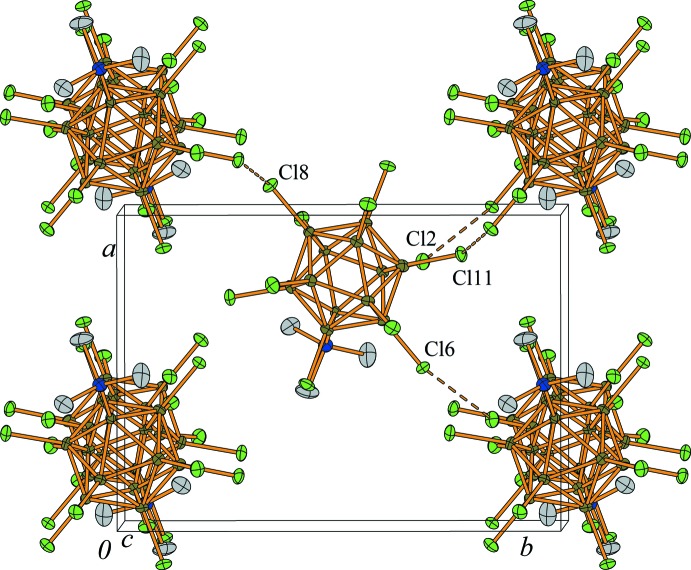
Part of the crystal structure illustrating the distorted body-centered cubic arrangement of the [Me_3_NB_12_Cl_11_]^−^ anions. Displacement ellipsoids are drawn at the 50% probability level and hydrogen atoms were omitted for clarity. Selected inter­molecular contacts below 3.5 Å are shown [dashed lines; Cl2⋯Cl6 = 3.492 (14) Å and Cl8⋯Cl11 = 3.3760 (14) Å].

The Na^+^ cation is surrounded by seven chlorine atoms from three different boron clusters and one oxygen atom from a SO_2_ mol­ecule, resulting in a total coordination number of 8 (Fig. 1 ▸) and giving rise to a three-dimensional network. The polyhedron around Na^+^ may be best described as a distorted twofold-capped trigonal prism (Fig. 3 ▸). The structure of the title compound is reminiscent of that of Ag[Me_3_NB_12_Cl_11_]·SO_2_ (Jenne & Wegener, 2018 ▸), although the coordination sphere around the metal cations is different in the two structures. The Na^+^⋯Cl contacts are weaker than the Ag^+^⋯Cl contacts and there is also only one SO_2_ mol­ecule per cation present in the title compound. The [Me_3_NB_12_Cl_11_]^−^ anions are placed in a body-centered cubic arrangement (Fig. 4 ▸) with some of the inter­molecular Cl⋯Cl distances being shorter than the sum of the van Waals radii (3.50 Å; Mantina *et al.*, 2009 ▸). The [Me_3_NB_12_Cl_11_]^−^ anions pack quite efficiently in the solid state and unlike in Na_2_[B_12_Cl_12_]·4SO_2_, where the structure contains two mol­ecules of SO_2_ per sodium cation to separate the doubly charged anions, only one SO_2_ mol­ecule is required in this case.

## Database survey   

The [Me_3_NB_12_Cl_11_]^−^ anion was first reported in 2014 (Bolli *et al.*, 2014 ▸) and a variety of crystal structures containing this anion have been published (*e.g*. Saleh *et al.*, 2016 ▸; Bertocco *et al.*, 2016 ▸; Bolli *et al.*, 2017 ▸; Jenne & Wegener, 2018 ▸), in which the [Me_3_NB_12_Cl_11_]^−^ anion is essentially identical to that reported in this study. Sodium complexes of fluorinated *closo*-dodeca­borates were studied recently by Strauss and co-workers (Bukovsky *et al.*, 2017**a* ▸,b* ▸). Sodium–SO_2_ complexes are still rare. Only the complex [Na(OSO)_1.5_][AlCl_4_] (Peters *et al.*, 1982 ▸) and four complexes of the type Na_2_[B_12_ *X*_12_]·*n*SO_2_ (*X* = H, Cl–I) (Derendorf *et al.*, 2010 ▸) are known. The number of *η* ^1^-O-bonded SO_2_ complexes is growing, although there is still some *terra incognita* in the Periodic Table. Structures published before the year 2000 are compiled in a review (Mews *et al.*, 2000 ▸). Recent examples include alkali-metal (Cameron *et al.*, 2010 ▸; Derendorf *et al.*, 2010 ▸; Malischewski *et al.*, 2016 ▸) and transition-metal complexes (Knapp & Mews, 2005 ▸; Akkuş *et al.*, 2006 ▸; Decken *et al.*, 2009 ▸; Aris *et al.*, 2011 ▸; Malischewski *et al.*, 2016 ▸, Jenne & Wegener, 2018 ▸).

## Synthesis and crystallization   

The crystals were obtained as a by-product from a reaction of [CPh_3_][Me_3_NB_12_Cl_11_] with Et_3_SiH and SO_2_ in 1,2-di­fluoro­benzene designed to give [Et_3_SiOS(H)OSiEt_3_][Me_3_NB_12_Cl_11_]^−^ in analogy to a published procedure (Kessler *et al.*, 2010 ▸). Crystallization of the red–brown product from 1,2-di­fluoro­benzene/*n*-pentane yielded the title compound as colorless crystals. The source of the sodium cation remains uncertain, but it may arise from an incomplete conversion of Na[Me_3_NB_12_Cl_11_] to [CPh_3_][Me_3_NB_12_Cl_11_] (Bolli *et al.*, 2014 ▸).

## Refinement   

Crystal data, data collection and structure refinement details are summarized in Table 2 ▸. H atoms were placed in calculated positions and refined as riding with C—H = 0.96 Å and *U*_iso_(H) = 1.5*U*_eq_(C).

## Supplementary Material

Crystal structure: contains datablock(s) I. DOI: 10.1107/S2056989019004663/cq2030sup1.cif

Structure factors: contains datablock(s) I. DOI: 10.1107/S2056989019004663/cq2030Isup2.hkl

CCDC reference: 1908217

Additional supporting information:  crystallographic information; 3D view; checkCIF report

## Figures and Tables

**Table 1:** Selected geometric parameters (Å, °)

Cl4—Na1^i^	3.209 (2)	Cl5—B5	1.800 (5)
Cl5—Na1^ii^	3.050 (2)	Cl6—B6	1.801 (4)
Cl6—Na1^ii^	3.031 (2)	Cl7—B7	1.792 (5)
Cl7—Na1	3.179 (2)	Cl8—B8	1.783 (4)
Cl9—Na1^i^	2.975 (2)	Cl9—B9	1.803 (4)
Cl10—Na1^ii^	3.051 (2)	Cl10—B10	1.800 (5)
Cl12—Na1	2.870 (2)	Cl11—B11	1.779 (5)
S1—O1	1.428 (3)	Cl12—B12	1.796 (5)
S1—O2	1.412 (4)	N1—B1	1.600 (5)
Na1—O1	2.428 (4)	N1—C1	1.510 (5)
Cl2—B2	1.785 (5)	N1—C2	1.503 (5)
Cl3—B3	1.797 (4)	N1—C3	1.503 (6)
Cl4—B4	1.800 (5)		
			
O1—S1—O2	116.8 (2)	S1—O1—Na1	147.0 (2)

Symmetry codes: (i) 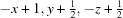; (ii) 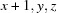.

**Table 2:** Experimental details

Crystal data	
Chemical formula	Na^+^C_3_H_9_B_12_Cl_11_N^−^·SO_2_
*M*_r_	665.83
Crystal system, space group	Orthorhombic, *P*2_1_2_1_2_1_
Temperature (K)	150
*a*, *b*, *c* (Å)	9.1943 (3), 12.9081 (4), 19.4486 (5)
*V* (Å^3^)	2308.19 (11)
*Z*	4
Radiation type	Mo *K*α
μ (mm^−1^)	1.44
Crystal size (mm)	0.06 × 0.05 × 0.05

Data collection	
Diffractometer	Rigaku Oxford Diffraction Xcalibur, Eos, Gemini ultra
Absorption correction	Multi-scan (*CrysAlis PRO*; Rigaku OD, 2015 ▸)
*T*_min_, *T*_max_	0.984, 1.000
No. of measured, independent and observed [*I* > 2σ(*I*)] reflections	10389, 4967, 4560
*R*_int_	0.032
(sin θ/λ)_max_ (Å^−1^)	0.639

Refinement	
*R*[*F* ^2^ > 2σ(*F* ^2^)], *wR*(*F* ^2^), *S*	0.031, 0.064, 1.04
No. of reflections	4967
No. of parameters	283
H-atom treatment	H-atom parameters constrained
Δρ_max_, Δρ_min_ (e Å^−3^)	0.32, −0.44
Absolute structure	Flack *x* determined using 1789 quotients [(*I* ^+^)−(*I* ^−^)]/[(*I* ^+^)+(*I* ^−^)] (Parsons *et al.*, 2013 ▸).
Absolute structure parameter	−0.09 (5)

Computer programs: *CrysAlis PRO* (Rigaku OD, 2015 ▸), *SHELXT* (Sheldrick, 2015*a* ▸), *SHELXL* (Sheldrick, 2015*b* ▸), *DIAMOND* (Brandenburg & Putz, 1999 ▸) and *OLEX2* (Dolomanov *et al.*, 2009 ▸).

## supplementary crystallographic information

### Crystal data

**Table:**

Na^+^·C_3_H_9_B_12_Cl_11_N^−^·SO_2_	*D*_x_ = 1.916 Mg m^−^^3^
*M**_r_* = 665.83	Mo *K*α radiation, λ = 0.71073 Å
Orthorhombic, *P*2_1_2_1_2_1_	Cell parameters from 4780 reflections
*a* = 9.1943 (3) Å	θ = 2.6–28.9°
*b* = 12.9081 (4) Å	µ = 1.44 mm^−^^1^
*c* = 19.4486 (5) Å	*T* = 150 K
*V* = 2308.19 (11) Å^3^	Block, colourless
*Z* = 4	0.06 × 0.05 × 0.05 mm
*F*(000) = 1296	

### Data collection

**Table:**

Rigaku Oxford Diffraction Xcalibur, Eos, Gemini ultra diffractometer	4967 independent reflections
Radiation source: fine-focus sealed X-ray tube, Enhance (Mo) X-ray Source	4560 reflections with *I* > 2σ(*I*)
Graphite monochromator	*R*_int_ = 0.032
Detector resolution: 16.2705 pixels mm^-1^	θ_max_ = 27.0°, θ_min_ = 1.9°
ω scans	*h* = −11→8
Absorption correction: multi-scan (CrysAlisPro; Rigaku OD, 2015)	*k* = −16→12
*T*_min_ = 0.984, *T*_max_ = 1.000	*l* = −24→24
10389 measured reflections	

### Refinement

**Table:**

Refinement on *F*^2^	Hydrogen site location: inferred from neighbouring sites
Least-squares matrix: full	H-atom parameters constrained
*R*[*F*^2^ > 2σ(*F*^2^)] = 0.031	*w* = 1/[σ^2^(*F*_o_^2^) + (0.026*P*)^2^] where *P* = (*F*_o_^2^ + 2*F*_c_^2^)/3
*wR*(*F*^2^) = 0.064	(Δ/σ)_max_ < 0.001
*S* = 1.04	Δρ_max_ = 0.32 e Å^−^^3^
4967 reflections	Δρ_min_ = −0.44 e Å^−^^3^
283 parameters	Absolute structure: Flack *x* determined using 1789 quotients [(*I*^+^)-(*I*^-^)]/[(*I*^+^)+(*I*^-^)] (Parsons *et al.*, 2013).
0 restraints	Absolute structure parameter: −0.09 (5)
Primary atom site location: structure-invariant direct methods	

### Special details

**Table:**

Geometry. All esds (except the esd in the dihedral angle between two l.s. planes) are estimated using the full covariance matrix. The cell esds are taken into account individually in the estimation of esds in distances, angles and torsion angles; correlations between esds in cell parameters are only used when they are defined by crystal symmetry. An approximate (isotropic) treatment of cell esds is used for estimating esds involving l.s. planes.

### Fractional atomic coordinates and isotropic or equivalent isotropic displacement parameters (Å^2^)

**Table:**

	*x*	*y*	*z*	*U*_iso_*/*U*_eq_	
Cl3	0.54490 (10)	0.60120 (8)	0.50279 (5)	0.0174 (2)	
Cl6	1.01248 (10)	0.32550 (8)	0.37747 (5)	0.0167 (2)	
Cl5	1.06356 (11)	0.58650 (8)	0.30581 (5)	0.0194 (2)	
Cl8	0.43371 (11)	0.66888 (8)	0.33691 (5)	0.0183 (2)	
Cl2	0.68060 (11)	0.32798 (8)	0.49788 (5)	0.0183 (2)	
Cl4	0.79016 (11)	0.76099 (7)	0.38771 (5)	0.0181 (2)	
Cl10	0.88045 (11)	0.39211 (9)	0.21212 (5)	0.0189 (2)	
Cl11	0.65146 (11)	0.23842 (8)	0.32551 (5)	0.0171 (2)	
Cl12	0.49042 (11)	0.44663 (8)	0.22120 (5)	0.0186 (2)	
Cl9	0.74597 (11)	0.66169 (8)	0.21672 (5)	0.0197 (2)	
Cl7	0.37526 (11)	0.40537 (8)	0.39531 (5)	0.0172 (2)	
S1	0.11196 (13)	0.57107 (9)	0.11604 (6)	0.0263 (3)	
Na1	0.19934 (19)	0.38485 (15)	0.25524 (9)	0.0273 (4)	
O1	0.1870 (3)	0.4912 (2)	0.15243 (16)	0.0274 (8)	
N1	0.9459 (4)	0.5473 (3)	0.49125 (17)	0.0160 (7)	
B1	0.8383 (5)	0.5239 (4)	0.4285 (2)	0.0108 (9)	
B6	0.8625 (5)	0.4148 (4)	0.3718 (2)	0.0112 (9)	
B10	0.7980 (5)	0.4478 (4)	0.2875 (2)	0.0127 (9)	
B7	0.5540 (5)	0.4545 (3)	0.3782 (2)	0.0122 (9)	
B2	0.7093 (5)	0.4170 (4)	0.4290 (2)	0.0118 (9)	
C1	0.8866 (5)	0.6276 (4)	0.5402 (2)	0.0263 (11)	
H1A	0.860583	0.688810	0.515050	0.039*	
H1B	0.959336	0.644482	0.573805	0.039*	
H1C	0.802058	0.600510	0.562979	0.039*	
B5	0.8909 (5)	0.5418 (4)	0.3378 (2)	0.0123 (9)	
B4	0.7569 (5)	0.6247 (3)	0.3761 (2)	0.0128 (9)	
B9	0.7332 (5)	0.5772 (3)	0.2902 (2)	0.0122 (9)	
B11	0.6889 (5)	0.3712 (4)	0.3426 (2)	0.0130 (10)	
B3	0.6441 (4)	0.5479 (4)	0.4315 (2)	0.0106 (9)	
C2	1.0899 (5)	0.5885 (4)	0.4670 (2)	0.0314 (12)	
H2A	1.134523	0.539081	0.436710	0.047*	
H2B	1.152153	0.600198	0.505865	0.047*	
H2C	1.075159	0.652549	0.442895	0.047*	
B8	0.5822 (5)	0.5814 (4)	0.3473 (2)	0.0117 (9)	
C3	0.9774 (6)	0.4535 (4)	0.5346 (3)	0.0359 (13)	
H3A	0.889899	0.431478	0.557199	0.054*	
H3B	1.049605	0.470500	0.568433	0.054*	
H3C	1.012930	0.398568	0.505844	0.054*	
B12	0.6101 (5)	0.4728 (3)	0.2921 (2)	0.0120 (9)	
O2	0.1730 (4)	0.6032 (3)	0.05292 (16)	0.0382 (9)	

### Atomic displacement parameters (Å^2^)

**Table:**

	*U*^11^	*U*^22^	*U*^33^	*U*^12^	*U*^13^	*U*^23^
Cl3	0.0191 (5)	0.0190 (5)	0.0140 (5)	0.0024 (4)	0.0047 (4)	−0.0039 (4)
Cl6	0.0121 (5)	0.0151 (5)	0.0231 (5)	0.0039 (4)	−0.0010 (4)	−0.0001 (4)
Cl5	0.0131 (5)	0.0220 (6)	0.0231 (5)	−0.0063 (4)	0.0040 (4)	−0.0007 (4)
Cl8	0.0164 (5)	0.0185 (5)	0.0200 (5)	0.0073 (4)	−0.0030 (4)	0.0016 (4)
Cl2	0.0239 (5)	0.0172 (5)	0.0138 (5)	−0.0002 (4)	0.0010 (4)	0.0054 (4)
Cl4	0.0229 (6)	0.0098 (5)	0.0217 (5)	−0.0018 (4)	0.0002 (4)	−0.0018 (4)
Cl10	0.0191 (5)	0.0234 (6)	0.0141 (5)	−0.0009 (4)	0.0052 (4)	−0.0054 (4)
Cl11	0.0212 (5)	0.0117 (5)	0.0183 (5)	−0.0027 (4)	−0.0042 (4)	−0.0031 (4)
Cl12	0.0170 (5)	0.0256 (6)	0.0131 (5)	−0.0022 (4)	−0.0049 (4)	−0.0010 (4)
Cl9	0.0252 (5)	0.0191 (6)	0.0149 (5)	−0.0024 (4)	0.0008 (4)	0.0070 (4)
Cl7	0.0099 (5)	0.0225 (5)	0.0193 (5)	−0.0037 (4)	0.0022 (4)	0.0002 (5)
S1	0.0254 (6)	0.0214 (6)	0.0322 (7)	0.0024 (5)	−0.0012 (5)	0.0017 (5)
Na1	0.0230 (9)	0.0285 (11)	0.0303 (10)	0.0011 (8)	0.0057 (8)	0.0049 (8)
O1	0.0253 (17)	0.0265 (19)	0.0303 (19)	−0.0009 (15)	−0.0034 (15)	0.0026 (15)
N1	0.0158 (17)	0.0180 (19)	0.0140 (18)	0.0008 (15)	−0.0060 (15)	−0.0035 (15)
B1	0.011 (2)	0.009 (2)	0.012 (2)	0.0009 (17)	−0.0018 (19)	−0.0025 (18)
B6	0.010 (2)	0.013 (2)	0.010 (2)	0.0026 (18)	0.0006 (18)	−0.0012 (18)
B10	0.009 (2)	0.016 (2)	0.013 (2)	−0.0010 (18)	−0.0003 (18)	−0.0010 (19)
B7	0.008 (2)	0.015 (2)	0.014 (2)	−0.0011 (18)	−0.0003 (19)	0.0004 (18)
B2	0.013 (2)	0.013 (2)	0.009 (2)	−0.0019 (18)	−0.0010 (18)	0.0008 (18)
C1	0.027 (2)	0.032 (3)	0.020 (2)	0.005 (2)	−0.008 (2)	−0.012 (2)
B5	0.011 (2)	0.014 (2)	0.012 (2)	−0.0023 (18)	0.0009 (19)	−0.0007 (18)
B4	0.015 (2)	0.009 (2)	0.015 (2)	−0.0027 (18)	0.0004 (19)	0.0014 (17)
B9	0.013 (2)	0.014 (2)	0.009 (2)	−0.0013 (19)	0.0012 (18)	0.0028 (18)
B11	0.013 (2)	0.015 (2)	0.011 (2)	−0.0015 (18)	−0.0022 (18)	−0.0028 (18)
B3	0.009 (2)	0.012 (2)	0.011 (2)	0.0011 (18)	0.0007 (18)	0.0003 (18)
C2	0.017 (2)	0.049 (4)	0.029 (3)	−0.007 (2)	−0.005 (2)	−0.012 (2)
B8	0.011 (2)	0.011 (2)	0.013 (2)	−0.0001 (18)	−0.0029 (18)	0.0032 (18)
C3	0.054 (4)	0.026 (3)	0.028 (3)	0.001 (2)	−0.028 (2)	0.004 (2)
B12	0.013 (2)	0.014 (2)	0.009 (2)	−0.0008 (18)	−0.0004 (19)	0.0007 (18)
O2	0.049 (2)	0.041 (2)	0.0248 (19)	−0.0089 (19)	−0.0077 (16)	0.0012 (17)

### Geometric parameters (Å, º)

**Table:**

Cl4—Na1^i^	3.209 (2)	B6—B5	1.787 (6)
Cl5—Na1^ii^	3.050 (2)	B6—B11	1.785 (6)
Cl6—Na1^ii^	3.031 (2)	B10—B5	1.777 (6)
Cl7—Na1	3.179 (2)	B10—B9	1.774 (6)
Cl9—Na1^i^	2.975 (2)	B10—B11	1.770 (6)
Cl10—Na1^ii^	3.051 (2)	B10—B12	1.760 (6)
Cl12—Na1	2.870 (2)	B7—B2	1.802 (6)
S1—O1	1.428 (3)	B7—B11	1.782 (6)
S1—O2	1.412 (4)	B7—B3	1.792 (6)
Na1—O1	2.428 (4)	B7—B8	1.764 (6)
Cl2—B2	1.785 (5)	B7—B12	1.769 (6)
Cl3—B3	1.797 (4)	B2—B11	1.791 (6)
Cl4—B4	1.800 (5)	B2—B3	1.794 (7)
Cl5—B5	1.800 (5)	C1—H1A	0.9600
Cl6—B6	1.801 (4)	C1—H1B	0.9600
Cl7—B7	1.792 (5)	C1—H1C	0.9600
Cl8—B8	1.783 (4)	B5—B4	1.794 (6)
Cl9—B9	1.803 (4)	B5—B9	1.779 (6)
Cl10—B10	1.800 (5)	B4—B9	1.792 (6)
Cl11—B11	1.779 (5)	B4—B3	1.794 (6)
Cl12—B12	1.796 (5)	B4—B8	1.791 (6)
N1—B1	1.600 (5)	B9—B8	1.779 (6)
N1—C1	1.510 (5)	B9—B12	1.760 (6)
N1—C2	1.503 (5)	B11—B12	1.792 (7)
N1—C3	1.503 (6)	B3—B8	1.785 (6)
B1—B6	1.803 (6)	C2—H2A	0.9600
B1—B2	1.820 (6)	C2—H2B	0.9600
B1—B5	1.844 (7)	C2—H2C	0.9600
B1—B4	1.813 (6)	B8—B12	1.785 (6)
B1—B3	1.813 (6)	C3—H3A	0.9600
B6—B10	1.794 (6)	C3—H3B	0.9600
B6—B2	1.795 (6)	C3—H3C	0.9600
			
B6—Cl6—Na1^ii^	102.94 (15)	H1A—C1—H1B	109.5
B5—Cl5—Na1^ii^	101.48 (16)	H1A—C1—H1C	109.5
B4—Cl4—Na1^i^	112.56 (16)	H1B—C1—H1C	109.5
B10—Cl10—Na1^ii^	101.13 (15)	Cl5—B5—B1	127.0 (3)
B12—Cl12—Na1	116.54 (15)	B6—B5—Cl5	123.4 (3)
B9—Cl9—Na1^i^	116.86 (15)	B6—B5—B1	59.5 (2)
B7—Cl7—Na1	109.68 (16)	B6—B5—B4	107.0 (3)
O1—S1—O2	116.8 (2)	B10—B5—Cl5	116.9 (3)
Cl6^iii^—Na1—Cl5^iii^	74.39 (5)	B10—B5—B1	108.3 (3)
Cl6^iii^—Na1—Cl4^iv^	124.79 (7)	B10—B5—B6	60.4 (2)
Cl6^iii^—Na1—Cl10^iii^	71.26 (5)	B10—B5—B4	107.8 (3)
Cl6^iii^—Na1—Cl7	68.74 (4)	B10—B5—B9	59.8 (2)
Cl5^iii^—Na1—Cl4^iv^	135.81 (7)	B4—B5—Cl5	123.9 (3)
Cl5^iii^—Na1—Cl10^iii^	70.67 (5)	B4—B5—B1	59.8 (2)
Cl5^iii^—Na1—Cl7	82.00 (5)	B9—B5—Cl5	117.2 (3)
Cl10^iii^—Na1—Cl4^iv^	78.81 (5)	B9—B5—B1	108.4 (3)
Cl10^iii^—Na1—Cl7	136.21 (6)	B9—B5—B6	108.0 (3)
Cl12—Na1—Cl6^iii^	141.23 (7)	B9—B5—B4	60.2 (3)
Cl12—Na1—Cl5^iii^	102.65 (6)	Cl4—B4—B1	124.1 (3)
Cl12—Na1—Cl4^iv^	84.87 (5)	B5—B4—Cl4	121.2 (3)
Cl12—Na1—Cl10^iii^	145.50 (7)	B5—B4—B1	61.5 (3)
Cl12—Na1—Cl9^iv^	98.84 (6)	B5—B4—B3	108.5 (3)
Cl12—Na1—Cl7	72.55 (5)	B9—B4—Cl4	118.1 (3)
Cl9^iv^—Na1—Cl6^iii^	72.98 (5)	B9—B4—B1	109.1 (3)
Cl9^iv^—Na1—Cl5^iii^	146.78 (7)	B9—B4—B5	59.5 (3)
Cl9^iv^—Na1—Cl4^iv^	70.81 (5)	B9—B4—B3	107.4 (3)
Cl9^iv^—Na1—Cl10^iii^	104.04 (6)	B3—B4—Cl4	124.3 (3)
Cl9^iv^—Na1—Cl7	80.65 (5)	B3—B4—B1	60.3 (2)
Cl7—Na1—Cl4^iv^	140.24 (6)	B8—B4—Cl4	119.8 (3)
O1—Na1—Cl6^iii^	139.66 (10)	B8—B4—B1	108.8 (3)
O1—Na1—Cl5^iii^	76.35 (9)	B8—B4—B5	107.5 (3)
O1—Na1—Cl4^iv^	64.50 (9)	B8—B4—B9	59.5 (2)
O1—Na1—Cl10^iii^	73.23 (9)	B8—B4—B3	59.8 (2)
O1—Na1—Cl12	72.33 (9)	B10—B9—Cl9	121.6 (3)
O1—Na1—Cl9^iv^	134.94 (10)	B10—B9—B5	60.0 (3)
O1—Na1—Cl7	133.02 (10)	B10—B9—B4	108.0 (3)
S1—O1—Na1	147.0 (2)	B10—B9—B8	108.1 (3)
C1—N1—B1	112.8 (3)	B5—B9—Cl9	121.0 (3)
C2—N1—B1	111.8 (3)	B5—B9—B4	60.3 (3)
C2—N1—C1	105.9 (3)	B5—B9—B8	108.6 (3)
C2—N1—C3	106.9 (4)	B4—B9—Cl9	121.6 (3)
C3—N1—B1	113.3 (3)	B8—B9—Cl9	121.8 (3)
C3—N1—C1	105.6 (4)	B8—B9—B4	60.2 (2)
N1—B1—B6	122.5 (3)	B12—B9—Cl9	121.4 (3)
N1—B1—B2	122.8 (3)	B12—B9—B10	59.7 (2)
N1—B1—B5	123.0 (3)	B12—B9—B5	108.5 (3)
N1—B1—B4	123.3 (3)	B12—B9—B4	108.7 (3)
N1—B1—B3	123.5 (3)	B12—B9—B8	60.6 (2)
B6—B1—B2	59.4 (2)	Cl11—B11—B6	122.4 (3)
B6—B1—B5	58.7 (2)	Cl11—B11—B7	121.3 (3)
B6—B1—B4	105.5 (3)	Cl11—B11—B2	120.9 (3)
B6—B1—B3	105.9 (3)	Cl11—B11—B12	121.7 (3)
B2—B1—B5	105.7 (3)	B6—B11—B2	60.3 (2)
B4—B1—B2	106.2 (3)	B6—B11—B12	107.7 (3)
B4—B1—B5	58.7 (2)	B10—B11—Cl11	122.4 (3)
B3—B1—B2	59.2 (2)	B10—B11—B6	60.6 (2)
B3—B1—B5	105.5 (3)	B10—B11—B7	107.0 (3)
B3—B1—B4	59.3 (2)	B10—B11—B2	108.9 (3)
Cl6—B6—B1	123.8 (3)	B10—B11—B12	59.2 (2)
B10—B6—Cl6	117.5 (3)	B7—B11—B6	108.0 (3)
B10—B6—B1	109.4 (3)	B7—B11—B2	60.6 (3)
B10—B6—B2	107.7 (3)	B7—B11—B12	59.3 (3)
B2—B6—Cl6	124.9 (3)	B2—B11—B12	108.4 (3)
B2—B6—B1	60.8 (2)	Cl3—B3—B1	126.1 (3)
B5—B6—Cl6	119.9 (3)	B7—B3—Cl3	118.0 (3)
B5—B6—B1	61.8 (3)	B7—B3—B1	108.8 (3)
B5—B6—B10	59.5 (3)	B7—B3—B2	60.3 (2)
B5—B6—B2	109.2 (3)	B7—B3—B4	107.0 (3)
B11—B6—Cl6	120.2 (3)	B2—B3—Cl3	123.5 (3)
B11—B6—B1	109.3 (3)	B2—B3—B1	60.6 (2)
B11—B6—B10	59.3 (2)	B2—B3—B4	108.1 (3)
B11—B6—B2	60.1 (2)	B4—B3—Cl3	123.0 (3)
B11—B6—B5	107.6 (3)	B4—B3—B1	60.4 (2)
B6—B10—Cl10	120.7 (3)	B8—B3—Cl3	116.9 (3)
B5—B10—Cl10	121.2 (3)	B8—B3—B1	109.0 (3)
B5—B10—B6	60.1 (3)	B8—B3—B7	59.1 (3)
B9—B10—Cl10	122.8 (3)	B8—B3—B2	108.1 (3)
B9—B10—B6	107.9 (3)	B8—B3—B4	60.0 (2)
B9—B10—B5	60.1 (3)	N1—C2—H2A	109.5
B11—B10—Cl10	120.6 (3)	N1—C2—H2B	109.5
B11—B10—B6	60.1 (3)	N1—C2—H2C	109.5
B11—B10—B5	108.7 (3)	H2A—C2—H2B	109.5
B11—B10—B9	108.5 (3)	H2A—C2—H2C	109.5
B12—B10—Cl10	121.9 (3)	H2B—C2—H2C	109.5
B12—B10—B6	108.8 (3)	Cl8—B8—B4	121.7 (3)
B12—B10—B5	108.6 (3)	Cl8—B8—B3	120.1 (3)
B12—B10—B9	59.7 (3)	Cl8—B8—B12	122.6 (3)
B12—B10—B11	61.0 (3)	B7—B8—Cl8	121.0 (3)
Cl7—B7—B2	122.0 (3)	B7—B8—B4	108.4 (3)
B11—B7—Cl7	119.8 (3)	B7—B8—B9	107.4 (3)
B11—B7—B2	60.0 (3)	B7—B8—B3	60.6 (2)
B11—B7—B3	108.0 (3)	B7—B8—B12	59.8 (3)
B3—B7—Cl7	123.7 (3)	B9—B8—Cl8	123.1 (3)
B3—B7—B2	59.9 (2)	B9—B8—B4	60.3 (2)
B8—B7—Cl7	121.7 (3)	B9—B8—B3	108.4 (3)
B8—B7—B2	108.6 (3)	B9—B8—B12	59.2 (2)
B8—B7—B11	109.0 (3)	B3—B8—B4	60.2 (2)
B8—B7—B3	60.3 (2)	B12—B8—B4	107.7 (3)
B8—B7—B12	60.7 (3)	B12—B8—B3	108.4 (3)
B12—B7—Cl7	119.4 (3)	N1—C3—H3A	109.5
B12—B7—B2	108.9 (3)	N1—C3—H3B	109.5
B12—B7—B11	60.6 (3)	N1—C3—H3C	109.5
B12—B7—B3	108.8 (3)	H3A—C3—H3B	109.5
Cl2—B2—B1	126.1 (3)	H3A—C3—H3C	109.5
Cl2—B2—B6	124.9 (3)	H3B—C3—H3C	109.5
Cl2—B2—B7	117.8 (3)	B10—B12—Cl12	121.9 (3)
Cl2—B2—B11	118.5 (3)	B10—B12—B7	108.0 (3)
Cl2—B2—B3	122.5 (3)	B10—B12—B9	60.5 (3)
B6—B2—B1	59.8 (2)	B10—B12—B11	59.8 (3)
B6—B2—B7	106.6 (3)	B10—B12—B8	108.4 (3)
B7—B2—B1	108.0 (3)	B7—B12—Cl12	121.5 (3)
B11—B2—B1	108.3 (3)	B7—B12—B11	60.1 (3)
B11—B2—B6	59.7 (2)	B7—B12—B8	59.5 (3)
B11—B2—B7	59.5 (3)	B9—B12—Cl12	121.5 (3)
B11—B2—B3	107.5 (3)	B9—B12—B7	108.0 (3)
B3—B2—B1	60.2 (2)	B9—B12—B11	108.2 (3)
B3—B2—B6	107.1 (3)	B9—B12—B8	60.2 (3)
B3—B2—B7	59.8 (2)	B11—B12—Cl12	122.0 (3)
N1—C1—H1A	109.5	B8—B12—Cl12	121.4 (3)
N1—C1—H1B	109.5	B8—B12—B11	107.6 (3)
N1—C1—H1C	109.5		
			
Cl3—B3—B8—Cl8	2.8 (5)	B2—B1—B3—B4	−137.0 (3)
Cl3—B3—B8—B7	−108.0 (3)	B2—B1—B3—B8	−100.5 (3)
Cl3—B3—B8—B4	114.4 (3)	B2—B6—B10—Cl10	−146.9 (3)
Cl3—B3—B8—B9	152.0 (3)	B2—B6—B10—B5	102.3 (3)
Cl3—B3—B8—B12	−145.3 (3)	B2—B6—B10—B9	64.5 (4)
Cl6—B6—B10—Cl10	0.6 (5)	B2—B6—B10—B11	−37.0 (3)
Cl6—B6—B10—B5	−110.2 (3)	B2—B6—B10—B12	1.2 (4)
Cl6—B6—B10—B9	−148.0 (3)	B2—B6—B5—Cl5	155.9 (3)
Cl6—B6—B10—B11	110.5 (3)	B2—B6—B5—B1	39.3 (3)
Cl6—B6—B10—B12	148.7 (3)	B2—B6—B5—B10	−99.6 (3)
Cl6—B6—B2—Cl2	−2.3 (5)	B2—B6—B5—B4	1.5 (4)
Cl6—B6—B2—B1	112.8 (4)	B2—B6—B5—B9	−61.9 (4)
Cl6—B6—B2—B7	−145.7 (3)	B2—B6—B11—Cl11	−109.7 (4)
Cl6—B6—B2—B11	−107.8 (4)	B2—B6—B11—B10	138.6 (3)
Cl6—B6—B2—B3	151.6 (3)	B2—B6—B11—B7	38.8 (3)
Cl6—B6—B5—Cl5	1.8 (5)	B2—B6—B11—B12	101.4 (3)
Cl6—B6—B5—B1	−114.9 (3)	B2—B7—B11—Cl11	110.3 (4)
Cl6—B6—B5—B10	106.2 (3)	B2—B7—B11—B6	−38.6 (3)
Cl6—B6—B5—B4	−152.6 (3)	B2—B7—B11—B10	−102.5 (3)
Cl6—B6—B5—B9	143.9 (3)	B2—B7—B11—B12	−139.0 (3)
Cl6—B6—B11—Cl11	5.7 (5)	B2—B7—B3—Cl3	−114.6 (3)
Cl6—B6—B11—B10	−106.0 (3)	B2—B7—B3—B1	37.8 (3)
Cl6—B6—B11—B7	154.2 (3)	B2—B7—B3—B4	101.6 (3)
Cl6—B6—B11—B2	115.4 (3)	B2—B7—B3—B8	139.2 (3)
Cl6—B6—B11—B12	−143.1 (3)	B2—B7—B8—Cl8	−146.1 (3)
Cl5—B5—B4—Cl4	−2.0 (5)	B2—B7—B8—B4	1.5 (4)
Cl5—B5—B4—B1	−116.6 (4)	B2—B7—B8—B9	65.1 (4)
Cl5—B5—B4—B9	104.5 (4)	B2—B7—B8—B3	−36.6 (3)
Cl5—B5—B4—B3	−155.8 (3)	B2—B7—B8—B12	101.7 (3)
Cl5—B5—B4—B8	141.1 (3)	B2—B7—B12—Cl12	148.3 (3)
Cl5—B5—B9—Cl9	−4.2 (5)	B2—B7—B12—B10	−0.1 (4)
Cl5—B5—B9—B10	106.8 (3)	B2—B7—B12—B9	−64.1 (4)
Cl5—B5—B9—B4	−115.4 (3)	B2—B7—B12—B11	36.9 (3)
Cl5—B5—B9—B8	−152.6 (3)	B2—B7—B12—B8	−101.3 (3)
Cl5—B5—B9—B12	143.1 (3)	B2—B11—B12—Cl12	−147.6 (3)
Cl8—B8—B12—Cl12	1.0 (5)	B2—B11—B12—B10	101.5 (3)
Cl8—B8—B12—B10	149.9 (3)	B2—B11—B12—B7	−37.0 (3)
Cl8—B8—B12—B7	−109.5 (4)	B2—B11—B12—B9	63.7 (4)
Cl8—B8—B12—B9	111.9 (4)	B2—B11—B12—B8	0.1 (4)
Cl8—B8—B12—B11	−146.9 (3)	B2—B3—B8—Cl8	147.5 (3)
Cl2—B2—B11—Cl11	−3.7 (5)	B2—B3—B8—B7	36.7 (3)
Cl2—B2—B11—B6	−115.9 (3)	B2—B3—B8—B4	−100.9 (3)
Cl2—B2—B11—B10	−153.4 (3)	B2—B3—B8—B9	−63.3 (4)
Cl2—B2—B11—B7	107.3 (3)	B2—B3—B8—B12	−0.6 (4)
Cl2—B2—B11—B12	143.8 (3)	C1—N1—B1—B6	−167.5 (4)
Cl2—B2—B3—Cl3	0.1 (5)	C1—N1—B1—B2	−95.3 (4)
Cl2—B2—B3—B1	116.1 (4)	C1—N1—B1—B5	121.3 (4)
Cl2—B2—B3—B7	−105.7 (4)	C1—N1—B1—B4	49.6 (5)
Cl2—B2—B3—B4	154.7 (3)	C1—N1—B1—B3	−23.1 (5)
Cl2—B2—B3—B8	−141.8 (3)	B5—B1—B6—Cl6	108.7 (4)
Cl4—B4—B9—Cl9	1.4 (5)	B5—B1—B6—B10	−36.9 (3)
Cl4—B4—B9—B10	149.3 (3)	B5—B1—B6—B2	−136.8 (3)
Cl4—B4—B9—B5	111.5 (3)	B5—B1—B6—B11	−100.1 (3)
Cl4—B4—B9—B8	−109.8 (3)	B5—B1—B2—Cl2	150.7 (3)
Cl4—B4—B9—B12	−147.4 (3)	B5—B1—B2—B6	37.4 (3)
Cl4—B4—B3—Cl3	2.9 (5)	B5—B1—B2—B7	−61.7 (4)
Cl4—B4—B3—B1	−113.1 (4)	B5—B1—B2—B11	1.2 (4)
Cl4—B4—B3—B7	144.6 (3)	B5—B1—B2—B3	−99.0 (3)
Cl4—B4—B3—B2	−151.8 (3)	B5—B1—B4—Cl4	−110.1 (4)
Cl4—B4—B3—B8	107.3 (4)	B5—B1—B4—B9	36.8 (3)
Cl4—B4—B8—Cl8	−5.6 (5)	B5—B1—B4—B3	136.5 (3)
Cl4—B4—B8—B7	−153.0 (3)	B5—B1—B4—B8	100.2 (3)
Cl4—B4—B8—B9	107.1 (3)	B5—B1—B3—Cl3	−148.8 (3)
Cl4—B4—B8—B3	−114.7 (4)	B5—B1—B3—B7	61.6 (4)
Cl4—B4—B8—B12	143.8 (3)	B5—B1—B3—B2	99.3 (3)
Cl10—B10—B5—Cl5	5.2 (5)	B5—B1—B3—B4	−37.7 (3)
Cl10—B10—B5—B1	−146.4 (3)	B5—B1—B3—B8	−1.2 (4)
Cl10—B10—B5—B6	−109.8 (4)	B5—B6—B10—Cl10	110.7 (3)
Cl10—B10—B5—B4	150.3 (3)	B5—B6—B10—B9	−37.8 (3)
Cl10—B10—B5—B9	112.5 (4)	B5—B6—B10—B11	−139.3 (3)
Cl10—B10—B9—Cl9	0.1 (5)	B5—B6—B10—B12	−101.1 (3)
Cl10—B10—B9—B5	−109.9 (4)	B5—B6—B2—Cl2	−154.9 (3)
Cl10—B10—B9—B4	−147.8 (3)	B5—B6—B2—B1	−39.7 (3)
Cl10—B10—B9—B8	148.5 (3)	B5—B6—B2—B7	61.8 (4)
Cl10—B10—B9—B12	110.5 (4)	B5—B6—B2—B11	99.7 (3)
Cl10—B10—B11—Cl11	−1.7 (5)	B5—B6—B2—B3	−1.0 (4)
Cl10—B10—B11—B6	110.1 (3)	B5—B6—B11—Cl11	147.8 (3)
Cl10—B10—B11—B7	−148.5 (3)	B5—B6—B11—B10	36.1 (3)
Cl10—B10—B11—B2	147.5 (3)	B5—B6—B11—B7	−63.7 (4)
Cl10—B10—B11—B12	−111.9 (4)	B5—B6—B11—B2	−102.5 (3)
Cl10—B10—B12—Cl12	−1.3 (5)	B5—B6—B11—B12	−1.1 (4)
Cl10—B10—B12—B7	147.0 (3)	B5—B10—B9—Cl9	110.0 (4)
Cl10—B10—B12—B9	−112.1 (4)	B5—B10—B9—B4	−37.9 (3)
Cl10—B10—B12—B11	109.9 (4)	B5—B10—B9—B8	−101.6 (3)
Cl10—B10—B12—B8	−150.0 (3)	B5—B10—B9—B12	−139.5 (3)
Cl11—B11—B12—Cl12	−0.5 (5)	B5—B10—B11—Cl11	−148.4 (3)
Cl11—B11—B12—B10	−111.4 (4)	B5—B10—B11—B6	−36.6 (3)
Cl11—B11—B12—B7	110.1 (4)	B5—B10—B11—B7	64.8 (4)
Cl11—B11—B12—B9	−149.2 (3)	B5—B10—B11—B2	0.8 (4)
Cl11—B11—B12—B8	147.2 (3)	B5—B10—B11—B12	101.3 (3)
Cl9—B9—B8—Cl8	−0.4 (5)	B5—B10—B12—Cl12	147.3 (3)
Cl9—B9—B8—B7	147.6 (3)	B5—B10—B12—B7	−64.5 (4)
Cl9—B9—B8—B4	−110.8 (4)	B5—B10—B12—B9	36.4 (3)
Cl9—B9—B8—B3	−148.4 (3)	B5—B10—B12—B11	−101.6 (3)
Cl9—B9—B8—B12	110.7 (4)	B5—B10—B12—B8	−1.5 (4)
Cl9—B9—B12—Cl12	−0.7 (5)	B5—B4—B9—Cl9	−110.2 (4)
Cl9—B9—B12—B10	110.8 (4)	B5—B4—B9—B10	37.8 (3)
Cl9—B9—B12—B7	−148.2 (3)	B5—B4—B9—B8	138.7 (3)
Cl9—B9—B12—B11	148.2 (3)	B5—B4—B9—B12	101.1 (3)
Cl9—B9—B12—B8	−111.4 (4)	B5—B4—B3—Cl3	155.7 (3)
Cl7—B7—B2—Cl2	0.1 (5)	B5—B4—B3—B1	39.7 (3)
Cl7—B7—B2—B1	−150.6 (3)	B5—B4—B3—B7	−62.7 (4)
Cl7—B7—B2—B6	146.4 (3)	B5—B4—B3—B2	1.0 (4)
Cl7—B7—B2—B11	108.4 (4)	B5—B4—B3—B8	−99.9 (3)
Cl7—B7—B2—B3	−113.1 (4)	B5—B4—B8—Cl8	−149.3 (3)
Cl7—B7—B11—Cl11	−1.7 (5)	B5—B4—B8—B7	63.3 (4)
Cl7—B7—B11—B6	−150.6 (3)	B5—B4—B8—B9	−36.6 (3)
Cl7—B7—B11—B10	145.5 (3)	B5—B4—B8—B3	101.6 (3)
Cl7—B7—B11—B2	−112.0 (4)	B5—B4—B8—B12	0.1 (4)
Cl7—B7—B11—B12	109.0 (4)	B5—B9—B8—Cl8	147.7 (3)
Cl7—B7—B3—Cl3	−4.1 (5)	B5—B9—B8—B7	−64.4 (4)
Cl7—B7—B3—B1	148.3 (3)	B5—B9—B8—B4	37.2 (3)
Cl7—B7—B3—B2	110.5 (4)	B5—B9—B8—B3	−0.3 (4)
Cl7—B7—B3—B4	−148.0 (3)	B5—B9—B8—B12	−101.2 (3)
Cl7—B7—B3—B8	−110.3 (4)	B5—B9—B12—Cl12	−147.8 (3)
Cl7—B7—B8—Cl8	3.9 (5)	B5—B9—B12—B10	−36.4 (3)
Cl7—B7—B8—B4	151.5 (3)	B5—B9—B12—B7	64.6 (4)
Cl7—B7—B8—B9	−144.9 (3)	B5—B9—B12—B11	1.1 (4)
Cl7—B7—B8—B3	113.4 (4)	B5—B9—B12—B8	101.4 (3)
Cl7—B7—B8—B12	−108.3 (4)	B4—B1—B6—Cl6	145.6 (3)
Cl7—B7—B12—Cl12	1.7 (5)	B4—B1—B6—B10	0.0 (4)
Cl7—B7—B12—B10	−146.7 (3)	B4—B1—B6—B2	−99.9 (3)
Cl7—B7—B12—B9	149.3 (3)	B4—B1—B6—B5	36.9 (3)
Cl7—B7—B12—B11	−109.7 (4)	B4—B1—B6—B11	−63.2 (4)
Cl7—B7—B12—B8	112.1 (3)	B4—B1—B2—Cl2	−148.0 (3)
Na1^ii^—Cl6—B6—B1	−106.3 (3)	B4—B1—B2—B6	98.8 (3)
Na1^ii^—Cl6—B6—B10	36.8 (3)	B4—B1—B2—B7	−0.4 (4)
Na1^ii^—Cl6—B6—B2	178.1 (3)	B4—B1—B2—B11	62.5 (4)
Na1^ii^—Cl6—B6—B5	−32.1 (3)	B4—B1—B2—B3	−37.6 (3)
Na1^ii^—Cl6—B6—B11	105.4 (3)	B4—B1—B5—Cl5	111.7 (4)
Na1^ii^—Cl5—B5—B1	104.0 (3)	B4—B1—B5—B6	−137.4 (3)
Na1^ii^—Cl5—B5—B6	29.3 (4)	B4—B1—B5—B10	−100.4 (3)
Na1^ii^—Cl5—B5—B10	−41.6 (3)	B4—B1—B5—B9	−37.0 (3)
Na1^ii^—Cl5—B5—B4	179.4 (3)	B4—B1—B3—Cl3	−111.1 (4)
Na1^ii^—Cl5—B5—B9	−109.7 (3)	B4—B1—B3—B7	99.3 (3)
Na1^i^—Cl4—B4—B1	143.7 (3)	B4—B1—B3—B2	137.0 (3)
Na1^i^—Cl4—B4—B5	69.0 (3)	B4—B1—B3—B8	36.5 (3)
Na1^i^—Cl4—B4—B9	−0.5 (3)	B4—B5—B9—Cl9	111.2 (4)
Na1^i^—Cl4—B4—B3	−141.4 (3)	B4—B5—B9—B10	−137.7 (3)
Na1^i^—Cl4—B4—B8	−69.6 (3)	B4—B5—B9—B8	−37.2 (3)
Na1^ii^—Cl10—B10—B6	−37.0 (3)	B4—B5—B9—B12	−101.5 (3)
Na1^ii^—Cl10—B10—B5	34.4 (3)	B4—B9—B8—Cl8	110.4 (4)
Na1^ii^—Cl10—B10—B9	106.8 (3)	B4—B9—B8—B7	−101.6 (3)
Na1^ii^—Cl10—B10—B11	−108.1 (3)	B4—B9—B8—B3	−37.6 (3)
Na1^ii^—Cl10—B10—B12	179.1 (3)	B4—B9—B8—B12	−138.4 (3)
Na1—Cl12—B12—B10	147.4 (3)	B4—B9—B12—Cl12	148.2 (3)
Na1—Cl12—B12—B7	3.4 (4)	B4—B9—B12—B10	−100.4 (3)
Na1—Cl12—B12—B9	−139.9 (3)	B4—B9—B12—B7	0.6 (4)
Na1—Cl12—B12—B11	75.5 (3)	B4—B9—B12—B11	−62.9 (4)
Na1—Cl12—B12—B8	−67.8 (3)	B4—B9—B12—B8	37.4 (3)
Na1^i^—Cl9—B9—B10	−145.2 (3)	B4—B3—B8—Cl8	−111.5 (4)
Na1^i^—Cl9—B9—B5	−73.5 (3)	B4—B3—B8—B7	137.6 (3)
Na1^i^—Cl9—B9—B4	−1.5 (4)	B4—B3—B8—B9	37.6 (3)
Na1^i^—Cl9—B9—B8	70.7 (3)	B4—B3—B8—B12	100.3 (3)
Na1^i^—Cl9—B9—B12	143.3 (3)	B4—B8—B12—Cl12	−148.1 (3)
Na1—Cl7—B7—B2	−147.3 (3)	B4—B8—B12—B10	0.8 (4)
Na1—Cl7—B7—B11	−76.1 (3)	B4—B8—B12—B7	101.4 (3)
Na1—Cl7—B7—B3	139.9 (3)	B4—B8—B12—B9	−37.2 (3)
Na1—Cl7—B7—B8	66.7 (3)	B4—B8—B12—B11	64.0 (4)
Na1—Cl7—B7—B12	−5.1 (3)	B9—B10—B5—Cl5	−107.3 (3)
N1—B1—B6—Cl6	−2.8 (6)	B9—B10—B5—B1	101.1 (3)
N1—B1—B6—B10	−148.5 (3)	B9—B10—B5—B6	137.7 (3)
N1—B1—B6—B2	111.7 (4)	B9—B10—B5—B4	37.8 (3)
N1—B1—B6—B5	−111.5 (4)	B9—B10—B11—Cl11	147.8 (3)
N1—B1—B6—B11	148.3 (4)	B9—B10—B11—B6	−100.4 (3)
N1—B1—B2—Cl2	2.0 (5)	B9—B10—B11—B7	1.0 (4)
N1—B1—B2—B6	−111.2 (4)	B9—B10—B11—B2	−63.0 (4)
N1—B1—B2—B7	149.6 (3)	B9—B10—B11—B12	37.5 (3)
N1—B1—B2—B11	−147.5 (3)	B9—B10—B12—Cl12	110.8 (4)
N1—B1—B2—B3	112.4 (4)	B9—B10—B12—B7	−100.9 (3)
N1—B1—B5—Cl5	0.0 (6)	B9—B10—B12—B11	−138.0 (3)
N1—B1—B5—B6	110.8 (4)	B9—B10—B12—B8	−37.9 (3)
N1—B1—B5—B10	147.9 (3)	B9—B5—B4—Cl4	−106.4 (4)
N1—B1—B5—B4	−111.8 (4)	B9—B5—B4—B1	138.9 (3)
N1—B1—B5—B9	−148.7 (3)	B9—B5—B4—B3	99.8 (3)
N1—B1—B4—Cl4	1.1 (5)	B9—B5—B4—B8	36.6 (3)
N1—B1—B4—B5	111.3 (4)	B9—B4—B3—Cl3	−141.4 (3)
N1—B1—B4—B9	148.1 (4)	B9—B4—B3—B1	102.5 (3)
N1—B1—B4—B3	−112.3 (4)	B9—B4—B3—B7	0.2 (4)
N1—B1—B4—B8	−148.6 (4)	B9—B4—B3—B2	63.8 (4)
N1—B1—B3—Cl3	0.7 (6)	B9—B4—B3—B8	−37.0 (3)
N1—B1—B3—B7	−148.9 (4)	B9—B4—B8—Cl8	−112.8 (4)
N1—B1—B3—B2	−111.2 (4)	B9—B4—B8—B7	99.9 (3)
N1—B1—B3—B4	111.8 (4)	B9—B4—B8—B3	138.2 (3)
N1—B1—B3—B8	148.3 (4)	B9—B4—B8—B12	36.7 (3)
B1—B6—B10—Cl10	148.6 (3)	B9—B8—B12—Cl12	−110.9 (4)
B1—B6—B10—B5	37.9 (3)	B9—B8—B12—B10	38.0 (3)
B1—B6—B10—B9	0.1 (4)	B9—B8—B12—B7	138.6 (3)
B1—B6—B10—B11	−101.4 (3)	B9—B8—B12—B11	101.2 (3)
B1—B6—B10—B12	−63.2 (4)	B11—B6—B10—Cl10	−109.9 (4)
B1—B6—B2—Cl2	−115.1 (4)	B11—B6—B10—B5	139.3 (3)
B1—B6—B2—B7	101.5 (3)	B11—B6—B10—B9	101.5 (3)
B1—B6—B2—B11	139.4 (3)	B11—B6—B10—B12	38.2 (3)
B1—B6—B2—B3	38.8 (3)	B11—B6—B2—Cl2	105.4 (4)
B1—B6—B5—Cl5	116.7 (4)	B11—B6—B2—B1	−139.4 (3)
B1—B6—B5—B10	−138.9 (3)	B11—B6—B2—B7	−37.9 (3)
B1—B6—B5—B4	−37.7 (3)	B11—B6—B2—B3	−100.7 (3)
B1—B6—B5—B9	−101.2 (3)	B11—B6—B5—Cl5	−140.4 (3)
B1—B6—B11—Cl11	−146.7 (3)	B11—B6—B5—B1	102.9 (3)
B1—B6—B11—B10	101.6 (3)	B11—B6—B5—B10	−36.0 (3)
B1—B6—B11—B7	1.8 (4)	B11—B6—B5—B4	65.2 (4)
B1—B6—B11—B2	−37.0 (3)	B11—B6—B5—B9	1.7 (4)
B1—B6—B11—B12	64.5 (4)	B11—B10—B5—Cl5	151.6 (3)
B1—B2—B11—Cl11	148.5 (3)	B11—B10—B5—B1	0.0 (4)
B1—B2—B11—B6	36.3 (3)	B11—B10—B5—B6	36.6 (3)
B1—B2—B11—B10	−1.3 (4)	B11—B10—B5—B4	−63.2 (4)
B1—B2—B11—B7	−100.6 (3)	B11—B10—B5—B9	−101.1 (3)
B1—B2—B11—B12	−64.1 (4)	B11—B10—B9—Cl9	−148.5 (3)
B1—B2—B3—Cl3	−116.0 (4)	B11—B10—B9—B5	101.4 (3)
B1—B2—B3—B7	138.2 (3)	B11—B10—B9—B4	63.5 (4)
B1—B2—B3—B4	38.6 (3)	B11—B10—B9—B8	−0.1 (4)
B1—B2—B3—B8	102.1 (3)	B11—B10—B9—B12	−38.1 (3)
B1—B5—B4—Cl4	114.6 (4)	B11—B10—B12—Cl12	−111.1 (4)
B1—B5—B4—B9	−138.9 (3)	B11—B10—B12—B7	37.1 (3)
B1—B5—B4—B3	−39.1 (3)	B11—B10—B12—B9	138.0 (3)
B1—B5—B4—B8	−102.3 (3)	B11—B10—B12—B8	100.1 (3)
B1—B5—B9—Cl9	148.0 (3)	B11—B7—B2—Cl2	−108.3 (3)
B1—B5—B9—B10	−101.0 (3)	B11—B7—B2—B1	101.0 (3)
B1—B5—B9—B4	36.8 (3)	B11—B7—B2—B6	38.0 (3)
B1—B5—B9—B8	−0.4 (4)	B11—B7—B2—B3	138.4 (3)
B1—B5—B9—B12	−64.7 (4)	B11—B7—B3—Cl3	−151.7 (3)
B1—B4—B9—Cl9	−147.8 (3)	B11—B7—B3—B1	0.7 (4)
B1—B4—B9—B10	0.1 (4)	B11—B7—B3—B2	−37.1 (3)
B1—B4—B9—B5	−37.7 (3)	B11—B7—B3—B4	64.4 (4)
B1—B4—B9—B8	101.0 (3)	B11—B7—B3—B8	102.1 (3)
B1—B4—B9—B12	63.4 (4)	B11—B7—B8—Cl8	150.1 (3)
B1—B4—B3—Cl3	116.1 (4)	B11—B7—B8—B4	−62.3 (4)
B1—B4—B3—B7	−102.3 (3)	B11—B7—B8—B9	1.4 (4)
B1—B4—B3—B2	−38.7 (3)	B11—B7—B8—B3	−100.3 (3)
B1—B4—B3—B8	−139.5 (3)	B11—B7—B8—B12	37.9 (3)
B1—B4—B8—Cl8	145.6 (3)	B11—B7—B12—Cl12	111.4 (4)
B1—B4—B8—B7	−1.7 (4)	B11—B7—B12—B10	−37.0 (3)
B1—B4—B8—B9	−101.6 (3)	B11—B7—B12—B9	−101.0 (3)
B1—B4—B8—B3	36.6 (3)	B11—B7—B12—B8	−138.2 (3)
B1—B4—B8—B12	−64.9 (4)	B11—B2—B3—Cl3	142.5 (3)
B1—B3—B8—Cl8	−148.2 (3)	B11—B2—B3—B1	−101.4 (3)
B1—B3—B8—B7	101.0 (3)	B11—B2—B3—B7	36.8 (3)
B1—B3—B8—B4	−36.6 (3)	B11—B2—B3—B4	−62.8 (4)
B1—B3—B8—B9	1.0 (4)	B11—B2—B3—B8	0.7 (4)
B1—B3—B8—B12	63.7 (4)	B3—B1—B6—Cl6	−152.5 (3)
B6—B1—B2—Cl2	113.2 (4)	B3—B1—B6—B10	61.9 (4)
B6—B1—B2—B7	−99.1 (3)	B3—B1—B6—B2	−38.0 (3)
B6—B1—B2—B11	−36.2 (3)	B3—B1—B6—B5	98.8 (3)
B6—B1—B2—B3	−136.4 (3)	B3—B1—B6—B11	−1.4 (4)
B6—B1—B5—Cl5	−110.9 (4)	B3—B1—B2—Cl2	−110.4 (4)
B6—B1—B5—B10	37.0 (3)	B3—B1—B2—B6	136.4 (3)
B6—B1—B5—B4	137.4 (3)	B3—B1—B2—B7	37.3 (3)
B6—B1—B5—B9	100.4 (3)	B3—B1—B2—B11	100.2 (3)
B6—B1—B4—Cl4	−147.0 (3)	B3—B1—B5—Cl5	149.7 (3)
B6—B1—B4—B5	−36.9 (3)	B3—B1—B5—B6	−99.5 (3)
B6—B1—B4—B9	−0.1 (4)	B3—B1—B5—B10	−62.4 (4)
B6—B1—B4—B3	99.6 (3)	B3—B1—B5—B4	37.9 (3)
B6—B1—B4—B8	63.3 (4)	B3—B1—B5—B9	1.0 (4)
B6—B1—B3—Cl3	150.0 (3)	B3—B1—B4—Cl4	113.4 (4)
B6—B1—B3—B7	0.4 (4)	B3—B1—B4—B5	−136.5 (3)
B6—B1—B3—B2	38.1 (3)	B3—B1—B4—B9	−99.6 (3)
B6—B1—B3—B4	−98.9 (3)	B3—B1—B4—B8	−36.3 (3)
B6—B1—B3—B8	−62.4 (4)	B3—B7—B2—Cl2	113.2 (3)
B6—B10—B5—Cl5	115.0 (3)	B3—B7—B2—B1	−37.4 (3)
B6—B10—B5—B1	−36.6 (3)	B3—B7—B2—B6	−100.4 (3)
B6—B10—B5—B4	−99.9 (3)	B3—B7—B2—B11	−138.4 (3)
B6—B10—B5—B9	−137.7 (3)	B3—B7—B11—Cl11	147.4 (3)
B6—B10—B9—Cl9	147.8 (3)	B3—B7—B11—B6	−1.5 (4)
B6—B10—B9—B5	37.8 (3)	B3—B7—B11—B10	−65.4 (4)
B6—B10—B9—B4	−0.1 (4)	B3—B7—B11—B2	37.1 (3)
B6—B10—B9—B8	−63.8 (4)	B3—B7—B11—B12	−101.9 (3)
B6—B10—B9—B12	−101.7 (3)	B3—B7—B8—Cl8	−109.5 (3)
B6—B10—B11—Cl11	−111.8 (4)	B3—B7—B8—B4	38.1 (3)
B6—B10—B11—B7	101.4 (3)	B3—B7—B8—B9	101.7 (3)
B6—B10—B11—B2	37.4 (3)	B3—B7—B8—B12	138.3 (3)
B6—B10—B11—B12	138.0 (3)	B3—B7—B12—Cl12	−148.1 (3)
B6—B10—B12—Cl12	−149.0 (3)	B3—B7—B12—B10	63.6 (4)
B6—B10—B12—B7	−0.7 (4)	B3—B7—B12—B9	−0.5 (4)
B6—B10—B12—B9	100.2 (3)	B3—B7—B12—B11	100.5 (3)
B6—B10—B12—B11	−37.8 (3)	B3—B7—B12—B8	−37.6 (3)
B6—B10—B12—B8	62.3 (4)	B3—B2—B11—Cl11	−147.9 (3)
B6—B2—B11—Cl11	112.2 (4)	B3—B2—B11—B6	99.9 (3)
B6—B2—B11—B10	−37.5 (3)	B3—B2—B11—B10	62.4 (4)
B6—B2—B11—B7	−136.9 (3)	B3—B2—B11—B7	−37.0 (3)
B6—B2—B11—B12	−100.4 (3)	B3—B2—B11—B12	−0.5 (4)
B6—B2—B3—Cl3	−154.6 (3)	B3—B4—B9—Cl9	148.3 (3)
B6—B2—B3—B1	−38.6 (3)	B3—B4—B9—B10	−63.8 (4)
B6—B2—B3—B7	99.6 (3)	B3—B4—B9—B5	−101.6 (3)
B6—B2—B3—B4	0.0 (4)	B3—B4—B9—B8	37.1 (3)
B6—B2—B3—B8	63.5 (4)	B3—B4—B9—B12	−0.5 (4)
B6—B5—B4—Cl4	152.2 (3)	B3—B4—B8—Cl8	109.0 (4)
B6—B5—B4—B1	37.6 (3)	B3—B4—B8—B7	−38.3 (3)
B6—B5—B4—B9	−101.3 (3)	B3—B4—B8—B9	−138.2 (3)
B6—B5—B4—B3	−1.5 (4)	B3—B4—B8—B12	−101.5 (3)
B6—B5—B4—B8	−64.7 (4)	B3—B8—B12—Cl12	148.2 (3)
B6—B5—B9—Cl9	−149.1 (3)	B3—B8—B12—B10	−62.8 (4)
B6—B5—B9—B10	−38.0 (3)	B3—B8—B12—B7	37.7 (3)
B6—B5—B9—B4	99.7 (3)	B3—B8—B12—B9	−100.9 (3)
B6—B5—B9—B8	62.6 (4)	B3—B8—B12—B11	0.4 (4)
B6—B5—B9—B12	−1.7 (4)	C2—N1—B1—B6	73.4 (5)
B6—B11—B12—Cl12	148.7 (3)	C2—N1—B1—B2	145.5 (4)
B6—B11—B12—B10	37.8 (3)	C2—N1—B1—B5	2.1 (5)
B6—B11—B12—B7	−100.8 (3)	C2—N1—B1—B4	−69.6 (5)
B6—B11—B12—B9	0.0 (4)	C2—N1—B1—B3	−142.2 (4)
B6—B11—B12—B8	−63.7 (4)	B8—B7—B2—Cl2	150.0 (3)
B10—B6—B2—Cl2	142.1 (3)	B8—B7—B2—B1	−0.7 (4)
B10—B6—B2—B1	−102.8 (3)	B8—B7—B2—B6	−63.7 (4)
B10—B6—B2—B7	−1.3 (4)	B8—B7—B2—B11	−101.7 (3)
B10—B6—B2—B11	36.6 (3)	B8—B7—B2—B3	36.8 (3)
B10—B6—B2—B3	−64.0 (4)	B8—B7—B11—Cl11	−148.7 (3)
B10—B6—B5—Cl5	−104.4 (4)	B8—B7—B11—B6	62.4 (4)
B10—B6—B5—B1	138.9 (3)	B8—B7—B11—B10	−1.5 (4)
B10—B6—B5—B4	101.2 (3)	B8—B7—B11—B2	101.0 (3)
B10—B6—B5—B9	37.7 (3)	B8—B7—B11—B12	−38.0 (3)
B10—B6—B11—Cl11	111.7 (4)	B8—B7—B3—Cl3	106.2 (3)
B10—B6—B11—B7	−99.8 (3)	B8—B7—B3—B1	−101.4 (3)
B10—B6—B11—B2	−138.6 (3)	B8—B7—B3—B2	−139.2 (3)
B10—B6—B11—B12	−37.2 (3)	B8—B7—B3—B4	−37.7 (3)
B10—B5—B4—Cl4	−144.1 (3)	B8—B7—B12—Cl12	−110.4 (4)
B10—B5—B4—B1	101.3 (3)	B8—B7—B12—B10	101.2 (3)
B10—B5—B4—B9	−37.6 (3)	B8—B7—B12—B9	37.2 (3)
B10—B5—B4—B3	62.1 (4)	B8—B7—B12—B11	138.2 (3)
B10—B5—B4—B8	−1.0 (4)	B8—B4—B9—Cl9	111.1 (3)
B10—B5—B9—Cl9	−111.1 (4)	B8—B4—B9—B10	−100.9 (3)
B10—B5—B9—B4	137.7 (3)	B8—B4—B9—B5	−138.7 (3)
B10—B5—B9—B8	100.6 (3)	B8—B4—B9—B12	−37.6 (3)
B10—B5—B9—B12	36.3 (3)	B8—B4—B3—Cl3	−104.4 (4)
B10—B9—B8—Cl8	−148.7 (3)	B8—B4—B3—B1	139.5 (3)
B10—B9—B8—B7	−0.8 (4)	B8—B4—B3—B7	37.2 (3)
B10—B9—B8—B4	100.8 (3)	B8—B4—B3—B2	100.9 (3)
B10—B9—B8—B3	63.3 (4)	B8—B9—B12—Cl12	110.7 (4)
B10—B9—B8—B12	−37.6 (3)	B8—B9—B12—B10	−137.8 (3)
B10—B9—B12—Cl12	−111.4 (4)	B8—B9—B12—B7	−36.8 (3)
B10—B9—B12—B7	101.0 (3)	B8—B9—B12—B11	−100.4 (3)
B10—B9—B12—B11	37.5 (3)	C3—N1—B1—B6	−47.6 (5)
B10—B9—B12—B8	137.8 (3)	C3—N1—B1—B2	24.5 (5)
B10—B11—B12—Cl12	110.9 (4)	C3—N1—B1—B5	−118.9 (4)
B10—B11—B12—B7	−138.5 (3)	C3—N1—B1—B4	169.5 (4)
B10—B11—B12—B9	−37.8 (3)	C3—N1—B1—B3	96.8 (5)
B10—B11—B12—B8	−101.4 (3)	B12—B10—B5—Cl5	−143.6 (3)
B7—B2—B11—Cl11	−110.9 (4)	B12—B10—B5—B1	64.8 (4)
B7—B2—B11—B6	136.9 (3)	B12—B10—B5—B6	101.4 (3)
B7—B2—B11—B10	99.3 (4)	B12—B10—B5—B4	1.6 (4)
B7—B2—B11—B12	36.5 (3)	B12—B10—B5—B9	−36.3 (3)
B7—B2—B3—Cl3	105.7 (3)	B12—B10—B9—Cl9	−110.4 (4)
B7—B2—B3—B1	−138.2 (3)	B12—B10—B9—B5	139.5 (3)
B7—B2—B3—B4	−99.7 (3)	B12—B10—B9—B4	101.6 (3)
B7—B2—B3—B8	−36.1 (3)	B12—B10—B9—B8	38.0 (3)
B7—B11—B12—Cl12	−110.6 (4)	B12—B10—B11—Cl11	110.2 (4)
B7—B11—B12—B10	138.5 (3)	B12—B10—B11—B6	−138.0 (3)
B7—B11—B12—B9	100.8 (3)	B12—B10—B11—B7	−36.6 (3)
B7—B11—B12—B8	37.1 (3)	B12—B10—B11—B2	−100.6 (4)
B7—B3—B8—Cl8	110.9 (3)	B12—B7—B2—Cl2	−145.5 (3)
B7—B3—B8—B4	−137.6 (3)	B12—B7—B2—B1	63.8 (4)
B7—B3—B8—B9	−100.0 (3)	B12—B7—B2—B6	0.8 (4)
B7—B3—B8—B12	−37.3 (3)	B12—B7—B2—B11	−37.2 (3)
B7—B8—B12—Cl12	110.6 (4)	B12—B7—B2—B3	101.3 (3)
B7—B8—B12—B10	−100.5 (3)	B12—B7—B11—Cl11	−110.7 (4)
B7—B8—B12—B9	−138.6 (3)	B12—B7—B11—B6	100.4 (3)
B7—B8—B12—B11	−37.3 (3)	B12—B7—B11—B10	36.5 (3)
B2—B1—B6—Cl6	−114.5 (4)	B12—B7—B11—B2	139.0 (3)
B2—B1—B6—B10	99.9 (3)	B12—B7—B3—Cl3	144.0 (3)
B2—B1—B6—B5	136.8 (3)	B12—B7—B3—B1	−63.6 (4)
B2—B1—B6—B11	36.7 (3)	B12—B7—B3—B2	−101.4 (3)
B2—B1—B5—Cl5	−148.7 (3)	B12—B7—B3—B4	0.2 (4)
B2—B1—B5—B6	−37.8 (3)	B12—B7—B3—B8	37.8 (3)
B2—B1—B5—B10	−0.7 (4)	B12—B7—B8—Cl8	112.2 (4)
B2—B1—B5—B4	99.6 (3)	B12—B7—B8—B4	−100.2 (3)
B2—B1—B5—B9	62.7 (4)	B12—B7—B8—B9	−36.5 (3)
B2—B1—B4—Cl4	151.0 (3)	B12—B7—B8—B3	−138.3 (3)
B2—B1—B4—B5	−98.9 (3)	B12—B9—B8—Cl8	−111.1 (4)
B2—B1—B4—B9	−62.1 (4)	B12—B9—B8—B7	36.8 (3)
B2—B1—B4—B3	37.6 (3)	B12—B9—B8—B4	138.4 (3)
B2—B1—B4—B8	1.3 (4)	B12—B9—B8—B3	100.9 (3)
B2—B1—B3—Cl3	111.8 (4)	O2—S1—O1—Na1	176.9 (3)
B2—B1—B3—B7	−37.7 (3)		

Symmetry codes: (i) −*x*+1, *y*+1/2, −*z*+1/2; (ii) *x*+1, *y*, *z*; (iii) *x*−1, *y*, *z*; (iv) −*x*+1, *y*−1/2, −*z*+1/2.

## References

[bb1] Akkuş, Ö. N., Decken, A., Knapp, C. & Passmore, J. (2006). *J. Chem. Crystallogr.* **36**, 321–329.

[bb2] Aris, D., Beck, J., Decken, A., Dionne, I., Schmedt auf der Günne, J., Hoffbauer, W., Köchner, T., Krossing, I., Passmore, J., Rivard, E., Steden, F. & Wang, X. (2011). *Dalton Trans.* **40**, 5865–5880. 10.1039/c0dt01251c 21552624

[bb3] Batsanov, S. S. (2001). *Inorg. Mater.* **37**, 871–885.

[bb4] Bertocco, P., Bolli, C., Derendorf, J., Jenne, C., Klein, A. & Stirnat, K. (2016). *Chem. Eur. J.* **22**, 16032–16036. 10.1002/chem.201603924 27704626

[bb5] Bolli, C., Derendorf, J., Jenne, C. & Kessler, M. (2017). *Eur. J. Inorg. Chem.* pp. 4552–4558.

[bb6] Bolli, C., Derendorf, J., Jenne, C., Scherer, H., Sindlinger, C. P. & Wegener, B. (2014). *Chem. Eur. J.* **20**, 13783–13792. 10.1002/chem.201403625 25196859

[bb7] Bolli, C., Derendorf, J., Kessler, M., Knapp, C., Scherer, H., Schulz, C. & Warneke, J. (2010). *Angew. Chem. Int. Ed.* **49**, 3536–3538. 10.1002/anie.200906627 20544906

[bb8] Brandenburg, K. & Putz, H. (1999). *DIAMOND*. Crystal Impact GbR, Bonn, Germany.

[bb9] Bukovsky, E. V., Peryshkov, D. V., Wu, H., Zhou, W., Tang, W. S., Jones, W. M., Stavila, V., Udovic, T. J. & Strauss, S. H. (2017*a*). *Inorg. Chem.* **56**, 4369–4379. 10.1021/acs.inorgchem.6b02920 28383911

[bb10] Bukovsky, E. V., Pluntze, A. M. & Strauss, S. H. (2017*b*). *J. Fluor. Chem.* **203**, 90–98.

[bb11] Cameron, T. S., Nikiforov, G. B., Passmore, J. & Rautiainen, J. M. (2010). *Dalton Trans.* **39**, 2587–2596. 10.1039/b923291e 20179852

[bb12] Decken, A., Knapp, C., Nikiforov, G. B., Passmore, J., Rautiainen, J. M., Wang, X. & Zeng, X. (2009). *Chem. Eur. J.* **15**, 6504–6517. 10.1002/chem.200802498 19449357

[bb14] Derendorf, J., Kessler, M., Knapp, C., Rühle, M. & Schulz, C. (2010). *Dalton Trans.* **39**, 8671–8678. 10.1039/c0dt00521e 20717610

[bb15] Dolomanov, O. V., Bourhis, L. J., Gildea, R. J., Howard, J. A. K. & Puschmann, H. (2009). *J. Appl. Cryst.* **42**, 339–341.

[bb16] Grabowsky, S., Luger, P., Buschmann, J., Schneider, T., Schirmeister, T., Sobolev, A. N. & Jayatilaka, D. (2012). *Angew. Chem. Int. Ed.* **51**, 6776–6779. 10.1002/anie.201200745 22644673

[bb17] Holder, C. H. & Fink, M. (1981). *J. Chem. Phys.* **75**, 5323–5325.

[bb18] Jenne, C. & Wegener, B. (2018). *Z. Anorg. Allg. Chem.* **644**, 1123–1132.

[bb19] Kessler, M., Knapp, C., Sagawe, V., Scherer, H. & Uzun, R. (2010). *Inorg. Chem.* **49**, 5223–5230. 10.1021/ic100337k 20465275

[bb20] Knapp, C. (2013). *Comprehensive Inorganic Chemistry II* Vol. 1, edited by J. Reedijk & K. Poeppelmeier, pp. 651–679. Amsterdam: Elsevier.

[bb21] Knapp, C. & Mews, R. (2005). *Eur. J. Inorg. Chem.* pp. 3536–3542.

[bb22] Malischewski, M., Peryshkov, D. V., Bukovsky, E. V., Seppelt, K. & Strauss, S. H. (2016). *Inorg. Chem.* **55**, 12254–12262. 10.1021/acs.inorgchem.6b01980 27934406

[bb23] Mantina, M., Chamberlin, A. C., Valero, R., Cramer, C. J. & Truhlar, D. G. (2009). *J. Phys. Chem. A*, **113**, 5806–5812. 10.1021/jp8111556 PMC3658832 19382751

[bb24] Mews, R., Lork, E., Watson, P. G. & Görtler, B. (2000). *Coord. Chem. Rev.* **197**, 277–320.

[bb25] Mingos, D. M. P. (1978). *Transition Met. Chem.* **3**, 1–15.

[bb26] Parsons, S., Flack, H. D. & Wagner, T. (2013). *Acta Cryst.* B**69**, 249–259. 10.1107/S2052519213010014 PMC3661305 23719469

[bb27] Peters, K., Simon, A., Peters, E. M., Kühnl, H. & Koslowski, B. (1982). *Z. Anorg. Allg. Chem.* **492**, 7–14.

[bb28] Rigaku OD (2015). *CrysAlis PRO*. Rigaku Oxford Diffraction, Yarnton, England.

[bb29] Ryan, R. R., Kubas, G. J., Moody, D. C. & Eller, P. G. (1981). *Struct. Bond.* **46**, 47–100.

[bb30] Saleh, M., Powell, D. R. & Wehmschulte, R. J. (2016). *Inorg. Chem.* **55**, 10617–10627. 10.1021/acs.inorgchem.6b01867 27704790

[bb31] Shannon, R. D. (1976). *Acta Cryst.* A**32**, 751–767.

[bb32] Sheldrick, G. M. (2015*a*). *Acta Cryst.* A**71**, 3–8.

[bb33] Sheldrick, G. M. (2015*b*). *Acta Cryst.* C**71**, 3–8.

[bb34] Simon, A., Peters, K., Peters, E. M., Kühnl, H. & Koslowski, B. (1980). *Z. Anorg. Allg. Chem.* **469**, 94–100.

[bb35] Waddington, T. C. (1965). *Non-Aqueous Solvent Systems.* London: Academic Press.

[bb36] Wegener, M., Huber, F., Bolli, C., Jenne, C. & Kirsch, S. F. (2015). *Chem. Eur. J.* **21**, 1328–1336. 10.1002/chem.201404487 25394284

